# Immunometabolic reprogramming in sepsis: mechanisms, clinical endotypes, and therapeutic opportunities

**DOI:** 10.3389/fimmu.2026.1731995

**Published:** 2026-03-30

**Authors:** Chibo Liu, Yanqun Cai, Qinfei Ma, Wei Sun

**Affiliations:** 1 College of Basic Medical Sciences, Medical Basic Research Innovation Center of Airway Disease in North China, Key Laboratory of Pathobiology, Ministry of Education, Jilin University, Changchun, China; 2 Jilin Province Cross-regional Cooperation Science and Technology Innovation Center of Aquatic Laboratory Animals, Jilin Provincial Science and Technology Agency, Changchun, China; 3 Jilin Province Zebrafish Genetic Engineering Laboratory, Jilin Province Development and Reform Commission, Changchun, China; 4 Department of Clinical Laboratory, Municipal Hospital Affiliated to Taizhou University, Zhejiang, Taizhou, China

**Keywords:** macrophages, metabolic reprogramming, sepsis, short-chain fatty acids, T cells

## Abstract

Sepsis, a systemic inflammatory syndrome triggered by infection, is tightly linked to dysregulated host immunometabolism. We review three hallmark metabolic alterations. First, a shift from oxidative phosphorylation (OXPHOS) to glycolysis provides rapid ATP early on; prolonged glycolytic engagement, however, drives excessive cytokine release through abnormal accumulation of metabolic intermediates. Second, impaired fatty acid oxidation (FAO) and disrupted cholesterol homeostasis not only compromise energy supply but also amplify pro-inflammatory signaling. Third, mitochondrial dysfunction unleashes reactive oxygen species (ROS) and derails metabolic homeostasis, promoting multi-organ injury. Notably, short-chain fatty acids (SCFAs) derived from the gut microbiota fine tune pro-versus anti-inflammatory responses via epigenetic regulation of immune cells. We further discuss how metabolic reprogramming governs macrophage polarization and T cell exhaustion, and we summarize therapeutic strategies that target key metabolic nodes. This review provides an integrated perspective on the immunometabolic mechanisms of sepsis and offers a rationale for metabolism-based precision interventions.

## 1 Introduction

Despite recent advances in our understanding of the pathophysiology of sepsis, the syndrome remains a leading cause of morbidity and mortality worldwide. Each year, >30 million people develop sepsis, and case fatality rates for severe sepsis or septic shock still approach 50% in some countries and hover between 20% and 30% in others. Even in high income nations, the incidence remains high, accounting for ~5.3 million deaths annually (1). Sepsis is characterized by a dysregulated host immune response to invading pathogens that precipitates systemic infection and inflammation. Clinically, it manifests as fever or hypothermia, leukocytosis or leukopenia, hypertension or hypotension due to peripheral vasodilatation, disseminated intravascular coagulation, and progressive multi-organ injury/failure that can culminate in death (2). According to the Third International Consensus Definitions for Sepsis and Septic Shock (Sepsis-3), sepsis is defined as life-threatening organ dysfunction caused by a dysregulated host response to infection (3). Rising incidence, prolonged hospitalization, and frequent invasive procedures increase the risk of nosocomial infection, while the emergence of antimicrobial resistant pathogens presents fresh challenges for intensivists (4). Clinical and epidemiological evidence indicates that antimicrobial resistance has become a critical obstacle in the management of adult and neonatal sepsis worldwide (5).

During sepsis, profound metabolic alterations occur in immune and non-immune cells alike, ultimately driving organ dysfunction in the heart, lungs, kidneys, liver, and brain (6). Clinical and translational study of septic patients indicate that the underlying pathogenesis reflects a disordered host response in which excessive inflammation coexists with immune suppression (7). To meet heightened energetic demands, cellular metabolic pathways are reprogrammed. In the hyper-inflammatory phase, cells preferentially generate ATP through glycolysis—even under normoxic conditions—rather than the more efficient oxidative phosphorylation (OXPHOS) pathway (8). This aerobic glycolysis can be advantageous early on, supplying metabolic intermediates that support biosynthesis, growth, proliferation, and differentiation (9). However, as sepsis progresses, mitochondrial dysfunction prevents restoration of OXPHOS and metabolic homeostasis, precipitating organ failure (10).

Recent reviews have outlined mitochondrial dysfunction and HIF-driven immunometabolic changes or focused on macrophage-centered therapeutic targets in sepsis. However, they rarely synthesize glucose, lipid, and short-chain fatty-acid metabolism across both innate and adaptive immune compartments within a unified framework (11–13). Immunometabolism refers to the modulation of immune cell development and function by metabolic programs in health and disease (including autoimmunity, cancer, and infection). It provides a promising framework for targeting dysfunctional and dying immune cells in sepsis. This review therefore summarizes the rewiring of glucose, lipid, and short-chain fatty acid metabolism in sepsis, delineates how metabolic reprogramming in macrophages and T cells influences disease trajectory, and discusses emerging therapeutics that manipulate these pathways. By integrating current evidence, we aim to deepen appreciation of immunometabolic reprogramming in sepsis and highlight its therapeutic potential.

## 2 Sepsis: clinical impact and immunometabolic background

Current definitions portray sepsis as a dysregulated host response to infection that precipitates life threatening organ dysfunction. This immune reaction is highly complex and heterogeneous, with pro- and anti-inflammatory processes occurring simultaneously. Although an early, vigorous response is essential for pathogen clearance, the balance can tip toward either injurious hyper-inflammation or profound immunosuppression, both of which promote organ damage. Heterogeneity arises from age, infectious etiology, focus of infection, genetic background and therapeutic interventions.

**Figure 1:**
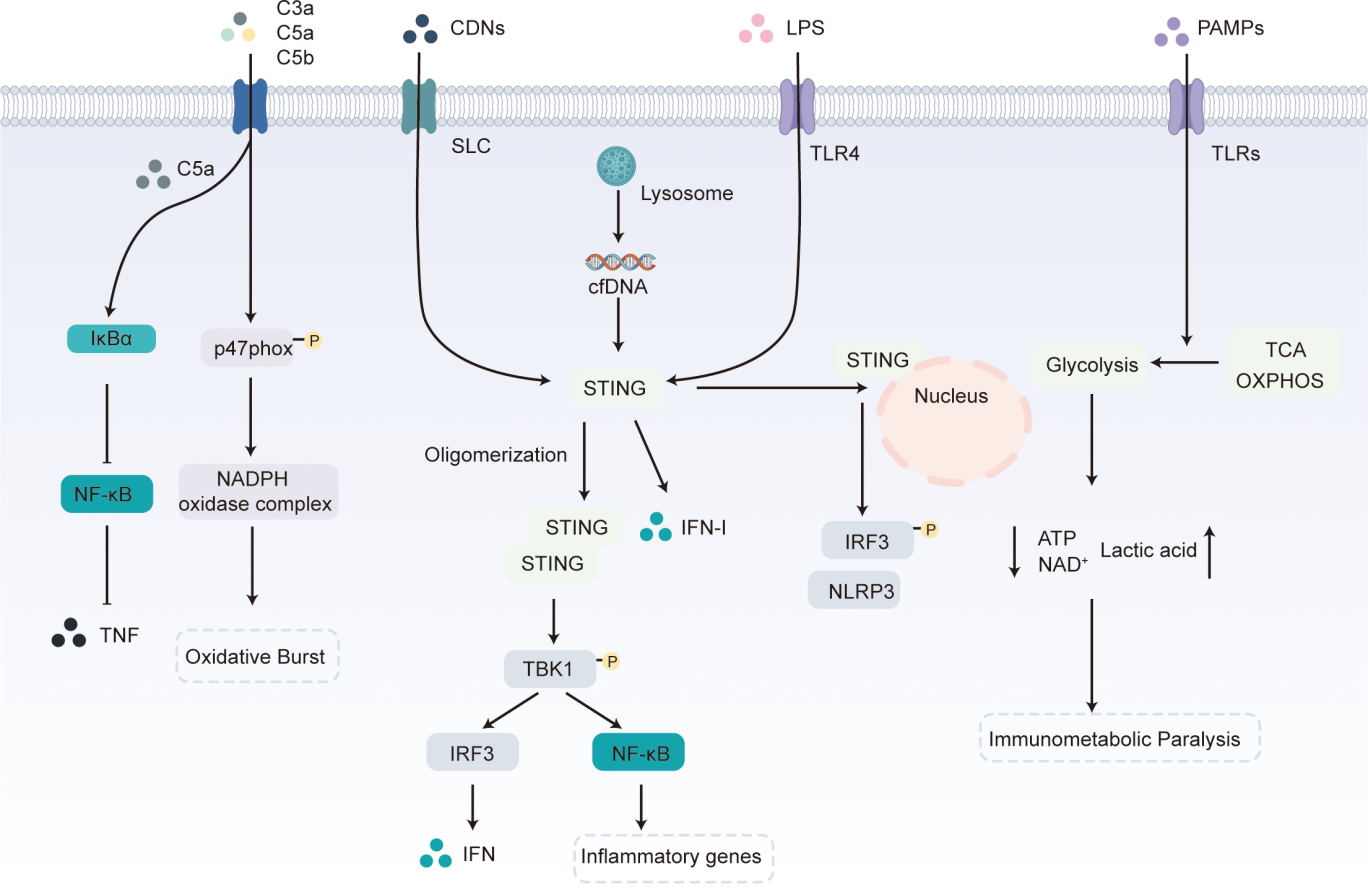
Innate-immune signaling and immunometabolic reprogramming. Binding of cyclic dinucleotides (CDNs) to STING promotes its homodimerization and subsequent higher order oligomerization, which facilitates phosphorylation of downstream TANK-binding kinase-1 (TBK1) dimers and activates both the IRF3 and NF-κB signaling pathways. Activated IRF3 drives the production of IFNs, whereas NF-κB regulates early inflammatory genes and endothelial cell surface molecule expression. Plasma cell-free DNA (cfDNA) that accumulates in lysosomes can likewise induce type I IFN production through a STING-dependent mechanism. Ligation of TLR4 by LPS causes perinuclear translocation of STING, followed by enhanced IRF3 phosphorylation and increased NLRP3 expression. Complement activation products C3a, C5a and C5b bind their membrane receptors to elicit pro-inflammatory responses; for example, C5a phosphorylates p47^phox, activates the NADPH oxidase complex and triggers an oxidative burst that kills pathogens. Excessive C5a, however, up-regulates IκBα, suppresses NF-κB mediated transcription and decreases tumor necrosis factor (TNF) secretion. Following PAMP stimulation, cells that normally generate energy via the tricarboxylic acid (TCA) cycle and OXPHOS shift toward aerobic glycolysis for rapid ATP supply, leading to ATP and NAD^+^ depletion, increased lactate production and the onset of “immunometabolic paralysis”.

The immune response in sepsis is initiated when pathogen-associated molecular patterns (PAMPs) engage pattern-recognition receptors (PRRs), triggering cascades such as cytokine and mediator release (14) (**Figure 1**). Bacterial cyclic dinucleotides (CDNs) that enter immune cells directly bind the stimulator of interferon genes (STING) protein, promoting its homodimerization and higher order oligomerization. Activated STING recruits and phosphorylates TANK binding kinase 1 (TBK1), which in turn activates interferon regulatory factor 3 (IRF3) and nuclear factor-κB (NF-κB) signaling (15, 16). IRF3 induces type I interferons (IFN-I), whereas NF-κB drives the transcription of early inflammatory genes and endothelial adhesion molecules (17). STING also senses apoptotic DNA. In sepsis, circulating cell-free DNA (cfDNA) rises markedly (18); undegraded apoptotic DNA accumulates in lysosomes and, via a STING-dependent mechanism, amplifies IFN-I production (19). Lipopolysaccharide (LPS) engagement of Toll-like receptor 4 (TLR4) relocates STING to the nuclear envelope, enhancing IRF3 phosphorylation and NLRP3 expression; STING-deficient mice exhibit lower myocardial and serum cytokine levels and are protected from septic cardiomyopathy (20).

Excessive tumor necrosis factor (TNF) and interleukin-1β (IL-1β) drive tissue injury, while neutrophils exacerbate damage by releasing reactive oxygen species (ROS), proteases, and neutrophil extracellular traps (NETs) (21). Sepsis also activates the complement cascade (22). Complement fragments C3a, C5a, and C5b bind membrane receptors and initiate multifaceted pro-inflammatory signaling (23). C5a, for instance, phosphorylates and translocates dormant p47^phox, thereby assembling the NADPH oxidase complex for pathogen-killing oxidative bursts (24). Sustained C5a overproduction, however, induces innate immune paralysis, most evident in neutrophils. It upregulates IκBα, suppresses NF-κB–dependent TNF release (25), impairing chemotaxis (26), and disrupts the C3a–C3aR axis to mobilize immature granulocytes and hematopoietic stem cells into the periphery, thereby fueling diffuse rather than targeted inflammation (27).

Under physiological conditions, anti-inflammatory circuits constrain exuberant inflammation. In ex vivo assays from clinical sepsis cohorts, peripheral monocytes from septic patients exhibit diminished cytokine output upon PAMP challenge (28). Clinical study similarly shows that, in septic patients, dendritic cells, natural killer cells, and neutrophils lose the capacity to produce key effector mediators essential for pathogen clearance (29). Mechanistic work in septic patients and relevant experimental models suggests that such innate defects reflect metabolic and epigenetic reprogramming sparked by the initial hyper-inflammatory insult (30). Peripheral monocytes in sepsis display a global metabolic paralysis—ATP and NAD^+^ depletion, lactate accumulation, and reduced oxygen consumption—termed “immunometabolic paralysis” (30). Adaptive immunity is likewise compromised (31): Clinical studies of septic shock patients demonstrate that T cell numbers fall through apoptosis, and surviving cells adopt an exhausted phenotype characterized by heightened checkpoint-molecule expression and blunted interferon-γ release (32). In these clinical cohorts, regulatory T cells (Tregs) become relatively enriched within the peripheral CD4^+^ T cell compartment (33).

Collectively, sepsis represents a severe disequilibrium of host immunity, oscillating between hyper-inflammation and immunosuppression. These divergent phenotypes are underpinned by immune cells metabolic reprogramming, and both correlate with poor outcomes. Deciphering the metabolic basis of immune dysfunction will facilitate patient enrichment and stratification in the clinic and may reveal novel targets for precision therapy.

## 3 Immunometabolic basis and global metabolic reprogramming

Metabolic dysfunction in immune and non-immune cells underlies numerous human diseases, including sepsis (34). During sepsis, obesity intensifies interactions between circulating cells and the endothelium and thereby aggravates inflammation (35). The mammalian target of rapamycin (mTOR) pathway, a key regulator of glycolysis and OXPHOS, drives metabolic reprogramming in CD4^+^ and CD8^+^ T cells; mTOR activation promotes a Warburg-like glycolytic program in CD4^+^ and CD8^+^ T cells, which has been linked to the early hyper-inflammatory state of sepsis (36, 37).

### 3.1 Integrative substrate flux remodeling in the septic milieu

#### 3.1.1 Lipid metabolic disruption

Post mortem analyses of patients who died from sepsis revealed intramyocellular lipid accumulation, likely resulting from disrupted fatty acid oxidation (FAO) and an imbalance between FAO and myocardial fatty acid uptake; these findings have been confirmed in experimental models (38). In immune cells, cholesterol accumulation activates pro-inflammatory Toll-like-receptor signaling (39). LPS stimulation promotes fatty acid and glucose uptake, enhances glucose to lipid conversion, upregulates triglyceride synthesis, and suppresses lipolysis, leading to triglyceride accumulation in macrophages (40).

**Figure 2:**
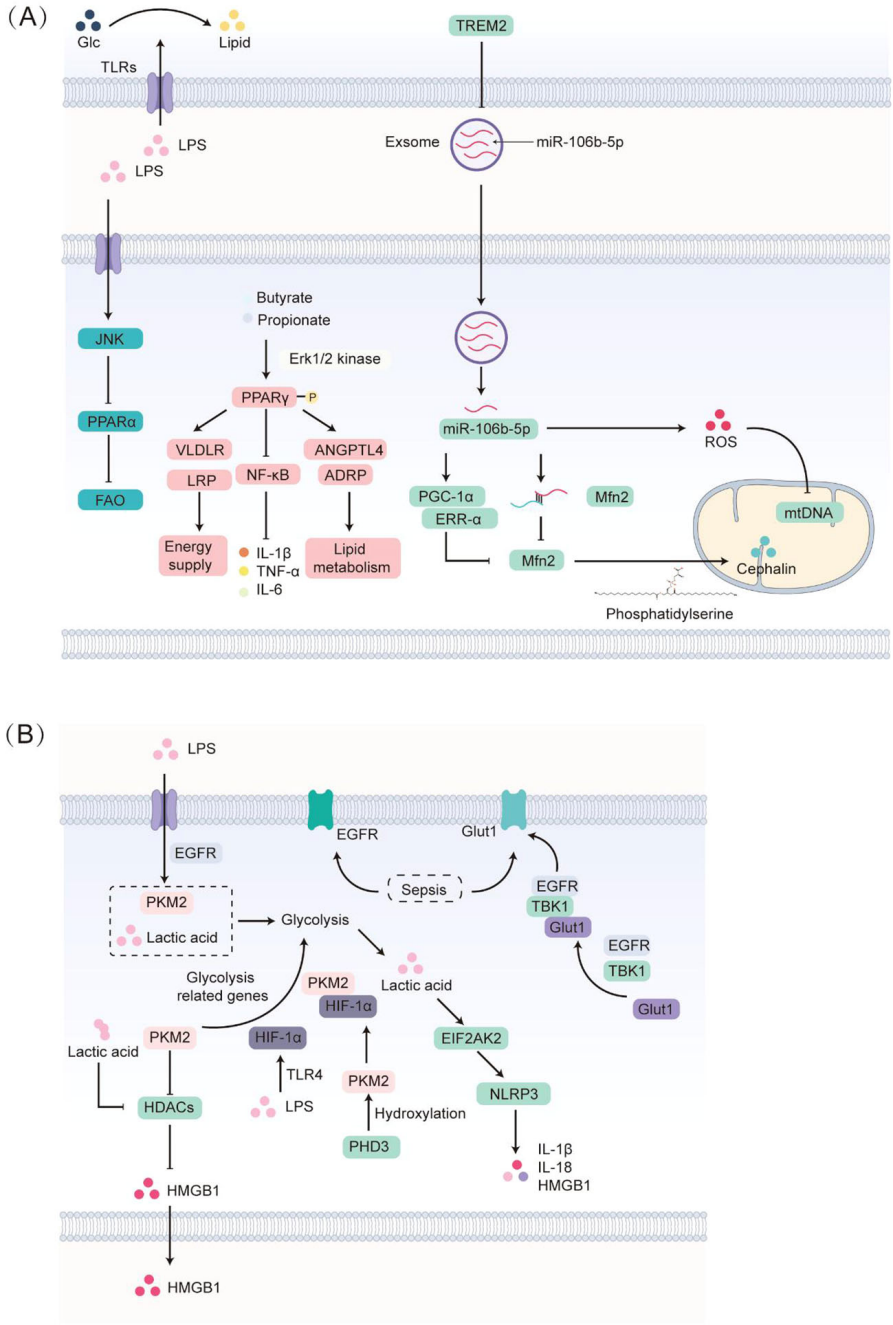
Lipid and glucose metabolic reprogramming of immune cells in sepsis. **(A)** Lipopolysaccharide (LPS) stimulation enhances fatty acid and glucose uptake, accelerates the conversion of glucose into lipids, up-regulates triglyceride synthesis and blocks its breakdown, leading to triglyceride accumulation in macrophages. Loss of TREM2 drives these cells to release exosomes enriched in miR-106b-5p; the microRNA targets the mitochondrial membrane protein mitofusin-2 (Mfn2) and lowers its abundance. miR-106b-5p also increases PGC-1α and ERR-α, further suppressing Mfn2. Because Mfn2 binds phosphatidylserine (PS) and promotes mitochondrial phosphatidylethanolamine (PE) biosynthesis, its inhibition compromises mitochondrial membranes. In parallel, miR-106b-5p boosts ROS production and reduces mitochondrial-DNA copy number, producing mitochondrial dysfunction. During sepsis, LPS acting through the JNK pathway down-regulates PPARα, so cells switch to PPARγ to govern energy metabolism. Activated PPARγ dampens NF-κB signaling and lowers IL-1β, TNF-α and IL-6. The short-chain fatty acids butyrate and propionate activate ERK1/2, cause PPARγ phosphorylation and activation, and induce its target genes ANGPTL4 and ADRP, thereby fine-tuning lipid handling. PPARγ activation also restores myocardial expression of the fatty acid uptake receptors VLDLR and LRP, improving energy supply. **(B)** Sepsis increases plasma-membrane expression of EGFR and Glut1. EGFR forms a complex with TBK1 and Glut1, moving Glut1 from the cytosol to the cell surface and, under LPS challenge, boosting glycolytic-enzyme levels, lactate production and phosphorylation of pyruvate kinase M2 (PKM2). PKM2 inhibits histone deacetylases (HDACs), restraining HMGB1 release, while LPS via TLR4 markedly elevates HIF-1α. HIF-1α binds PKM2, creating a positive-feedback loop that amplifies aerobic glycolysis genes; their interaction requires proline hydroxylation of PKM2 by PHD3. Excess lactate generated by PKM2 further suppresses HDAC activity, sharply increasing HMGB1 acetylation, after which acetylated HMGB1 translocates to the cytosol and is secreted. Moreover, PKM2 driven glycolysis promotes lactate dependent phosphorylation of EIF2AK2, facilitating inflammasome activation.

In preclinical models of sepsis-induced liver injury, up-regulation of triggering receptor expressed on myeloid cells 2 (TREM2) correlates with improved hepatic lipid metabolism, lower triglyceride levels, and attenuated liver–lung injury (41). In a non-alcoholic fatty liver disease (NAFLD)–sepsis mouse model, TREM2 deficiency accelerates early NAFLD progression and increases susceptibility to sepsis (41). TREM2 regulates lipid metabolism in adipose tissue macrophages (**Figure 2A**); Trem2 knockout broadly suppresses the lipid associated macrophage program, causing adipocyte hypertrophy, systemic hypercholesterolemia, fat accumulation, and glucose intolerance (42). Mechanistic studies show that Trem2^-^/^-^ macrophages release more exosomes enriched in miR-106b-5p (41, 43). miR-106b-5p targets the 3′ untranslated region of mitofusin 2 (Mfn2) and reduces its abundance (44). The microRNA also up-regulates PGC-1α and ERR-α, further lowering Mfn2 (44). Because Mfn2 binds phosphatidylserine (PS) and transfers PS to mitochondria for phosphatidylethanolamine synthesis (45), its loss impairs mitochondrial lipid handling. miR-106b-5p simultaneously increases ROS, reduces mitochondrial DNA (mtDNA) copy number, and damages mitochondrial function (44), leading to higher hepatic lipid content, fewer long chain acyl-carnitines, decreased ATP production, and enhanced mitochondrial fission (41).

Sepsis-induced FAO impairment involves peroxisome proliferator-activated receptor α (PPARα). Although cardiac performance initially rises after endotoxin challenge, it cannot be sustained within 24 h in PPARα-deficient mice (46). LPS suppresses myocardial PPARα and its FAO-related targets, limiting fatty-acid-derived ATP and causing cardiac dysfunction (47). PPARα down-regulation appears to rely on the c-Jun N-terminal kinase (JNK) pathway, as the JNK inhibitor SP600125 rescues PPARα expression and prevents LPS-induced energetic and mechanical failure (48). In pediatric sepsis, reduced leukocyte PPARα predicts a poor outcome (49), and PPARα knockout decreases survival in adult models (50). With PPARα suppressed, the host increasingly depends on PPARγ to govern energy metabolism. Activated by endogenous and synthetic ligands such as thiazolidinediones, PPARγ promotes cholesterol efflux in macrophages and suppresses inflammation by antagonizing NF-κB (51). This pathway involves formyl-peptide-receptor-like 1 and TLR4, NF-κB–dependent cyclo-oxygenase-2 up-regulation, and subsequent PPARγ activation (51). PPARγ agonists inhibit NF-κB signaling and lower IL-1β, TNF-α and IL-6 in rat sepsis (52), though PPARγ activation has also been linked to lymphocyte apoptosis, an effect mitigated by PPARγ antagonists (53). Short-chain fatty acids (SCFAs) such as butyrate and propionate activate ERK1/2 kinases, phosphorylate PPARγ, and induce its targets ANGPTL4 and ADRP, thereby modulating lipid metabolism (54). PPARγ activation can also restore myocardial fatty-acid uptake receptors VLDLR and LRP, improving energy supply (47).

Overall, this section highlights that sepsis disrupts lipid metabolism at several levels: impaired fatty acid oxidation and a mismatch between fatty acid uptake and oxidation cause intramyocellular lipid accumulation, while cholesterol loading and LPS-driven metabolic reprogramming in immune cells promote triglyceride deposition in macrophages under pro-inflammatory Toll-like-receptor signaling. In parallel, TREM2–miR-106b-5p–Mfn2 signaling and the shift from PPARα- to PPARγ-dependent control of energy metabolism link mitochondrial dysfunction, hepatic lipid accumulation, and altered inflammatory and cardiac responses to the broader pattern of lipid metabolic disruption in sepsis.

#### 3.1.2 Glycolytic shift in carbohydrate metabolism

In mouse models, the acute phase of sepsis is characterized by high-rate aerobic glycolysis with increased lactate production (**Figure 2B**) (55). Lymphocyte apoptosis is a major cause of immunosuppression and correlates with poor prognosis (56). Septic patients exhibit elevated membrane EGFR and Glut1 expression and increased CD4^+^ T cell activation; EGFR forms a complex with TBK1 and Glut1, promoting Glut1 translocation to the plasma membrane (56). The glycolytic inhibitor 2-deoxy-d-glucose and EGFR inhibitors decrease glycolysis related enzymes, lactate, pyruvate, fructose-1,6-bisphosphate, and phosphorylation of PKM2 and LDHA in LPS-treated CD4^+^ T cells (56). Phosphorylated PKM2 and lactate production are key markers of heightened glycolysis (57), suggesting that EGFR mediated Glut1 trafficking enhances glycolysis, weakens immune function, and contributes to immune cell exhaustion in sepsis (58).

Enhanced glycolysis promotes Pyruvate kinase M2 (PKM2) oligomerization and nuclear translocation, where it phosphorylates and activates STAT3, driving IL-1β and IL-6 production (59). PKM2 interacts with hypoxia inducible factor 1α (HIF-1α) and suppresses HMGB1 release by signaling to histone deacetylases; the PKM2 inhibitor shikonin lowers serum lactate and HMGB1 and protects mice from lethal endotoxemia and sepsis (60). LPS up-regulates HIF-1α via a TLR4-dependent pathway, coinciding with surges in TNF-α, IL-1, IL-4, IL-6 and IL-12, highlighting HIF-1α as a key determinant of the septic phenotype (61). Hypoxia, inflammation or infection prevent HIF-1α degradation; accumulated HIF-1α binds PKM2, driving aerobic-glycolysis genes and reinforcing the Warburg effect (62). This interaction requires PHD3-mediated hydroxylation of PKM2 at Pro-403/408; PHD3 knock-down limits PKM2 co-activator function, glucose uptake and lactate production (62). Excess lactate inhibits HDACs, elevates HMGB1 acetylation, and promotes its cytoplasmic translocation and extracellular release (60). PKM2 driven glycolysis also enhances EIF2AK2 phosphorylation, activating NLRP3 and AIM2 inflammasomes; inhibiting PKM2 or EIF2AK2 reduces IL-1β, IL-18 and HMGB1 release and protects mice from lethal (63). Another glycolytic enzyme, glyceraldehyde-3-phosphate dehydrogenase, represses TNF translation by binding TNF mRNA when glycolysis is limited (64); in T cells, non-glycolytic GAPDH similarly binds IFN-γ mRNA to inhibit its translation (65).

Lactate, a principal glycolytic product, exerts strong immunosuppressive effects. It polarizes macrophages toward an M2 phenotype via mTORC1 and HIF-1α pathways (66) and expands MDSCs *in vitro* (67). Lactate activates GPR81 and, through ARRB2, down-regulates TLR-induced NLRP3 inflammasome formation and IL-1β production (68). It also deactivates YAP via GPR81, blocking YAP–NF-κB interaction and nuclear entry, thus suppressing pro-inflammatory cytokines (69). Exported through MCT4, lactate triggers “stop migration” signals in T cells via SLC5A12 and SLC16A1, modulating cell motility and cytokine secretion (70).

This section shows that acute sepsis is accompanied by a metabolic shift from OXPHOS to glycolysis in immune cells, driven in part by EGFR–TBK1–Glut1 complexes that enhance Glut1 trafficking and glycolytic flux in CD4^+^ T cells. Glycolysis-associated enzymes such as PKM2 and GAPDH acquire non-metabolic signaling roles: PKM2 cooperates with HIF-1α and PHD3 to reinforce a Warburg-like program, promote lactate production and induce IL-1β, IL-6 and HMGB1, while GAPDH can bind TNF and IFN-γ mRNAs to restrain their translation when glycolysis is limited. The accumulated lactate itself acts as a potent immunomodulatory metabolite, shaping macrophage polarization and MDSC expansion and, via GPR81, YAP and specific transporters such as MCT4, SLC5A12 and SLC16A1, dampening inflammasome activation, pro-inflammatory cytokine production and T cell migration, thereby linking the glycolytic shift in sepsis to complex patterns of immune activation and immunosuppression.

#### 3.1.3 Short-chain fatty acids and epigenetic modulation

Beyond major metabolic routes, certain pathways and metabolites—particularly SCFAs—change markedly in sepsis (71). Elevated cytokines lead to enterocyte apoptosis and increased gut permeability (72). SCFAs exert pivotal immunomodulatory and anti-inflammatory functions (73). In CLP-induced rat sepsis, fecal SCFAs fall while hippocampal TNF-α, IL-1β and IL-6 rise (74). Exogenous SCFAs added to LPS-stimulated primary neutrophils suppress NF-κB activation, lower TNF-α and inducible-nitric-oxide-synthase, and inhibit HDAC activity (75). Butyrate similarly reduces nitric oxide, IL-6 and IL-12 in LPS-stimulated bone-marrow-derived macrophages (76).

Sepsis progression involves widespread alterations in phosphorylation, ubiquitylation, methylation and acylation (77, 78). Inhibiting HDAC3 suppresses NF-κB activation, curtails macrophage hyper-inflammation, and lessens LPS-induced endotoxemia and CLP-induced sepsis (79). SCFAs modulate lysine acetylation by inhibiting HDAC activity, thereby influencing inflammation (80). Pentanoate supplies acetyl-CoA and blocks HDACs, acetylating histone H4 at the IL-10 promoter and inducing lymphocyte IL-10 production. Acetate enhances p70 S6-kinase acetylation and rpS6 phosphorylation, promotes differentiation toward effector T cells and Tregs, and reduces anti-CD3–induced inflammation in an IL-10-dependent manner. mTOR abrogates this effect, implicating the mTOR–S6K pathway (80). In anti-CD3-activated CD4^+^ cells, butyrate increases histone acetylation at the IL-22 promoter, promotes HIF-1α binding, and enhances IL-22 production, protecting the host from inflammatory injury (81).

Beyond acetylation, methylation also shapes innate immune responses in sepsis. In LPS-injected mice, histone methyltransferase Ash1l promotes H3K4 methylation at the Tnfaip3 promoter and suppresses TNF-α and IL-6 expression (82). SCFAs likewise influence methylation. Acetate activates DNA methylation in regulatory-T-cell regions, boosting Treg proliferation (83). By contrast, butyrate reduces methylation at GPR41/43 promoters in bone-marrow-derived macrophages, lowering GPR41/43 expression and mitigating inflammation (76).

### 3.2 Mitochondrial network disruption and redox-driven bioenergetic failure

**Figure 3:**
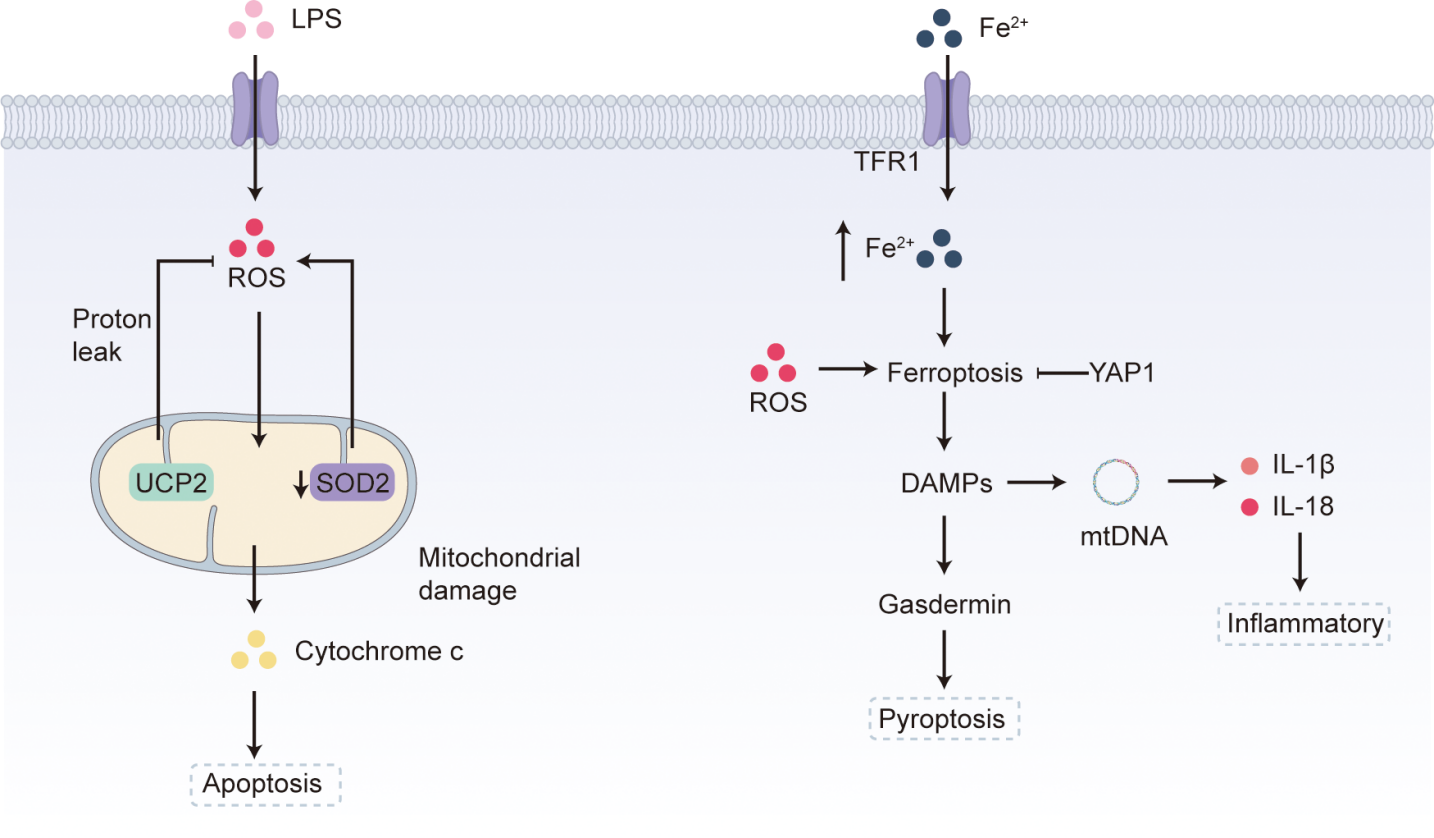
Mitochondrial metabolic reprogramming in sepsis. Lipopolysaccharide (LPS)–induced ROS production precipitates mitochondrial dysfunction. The uncoupling protein UCP2 limits ROS by permitting proton leak, yet during sepsis-associated apoptosis stressed or damaged mitochondria still release cytochrome c, driving lymphocyte and epithelial-cell death. Concomitant down-regulation of mitochondrial superoxide dismutase-2 (SOD2) amplifies ROS generation. Up-regulated transferrin-receptor-1 (TFR1) raises intracellular iron, and the combination of excess ROS and an expanded labile-iron pool triggers ferroptosis; dying cells then release damage-associated molecular patterns (DAMPs), further fueling inflammation. Inflammasome activation cleaves Gasdermin, causing membrane rupture and pyroptosis, whereas YAP1 can restrain ferroptotic progression. DAMPs also heighten ROS, inflicting additional mitochondrial injury and releasing mitochondrial DNA (mtDNA). Extracellular mtDNA enters neighboring cells, binds the NLRP3 inflammasome and promotes production of pro-inflammatory cytokines such as interleukin-1β (IL-1β) and IL-18, intensifying the systemic inflammatory response characteristic of sepsis.

Mitochondria regulate cellular metabolism and ATP production and are therefore pivotal during disease progression. They also control ROS generation, redox signaling and immune cells function. Timely, effective modulation of mitochondrial performance can improve sepsis outcomes and markedly lower mortality (58) (**Figure 3**). Hypoxia during sepsis induces glycolysis and suppresses OXPHOS, thereby reducing mitochondrial ROS and limiting self-inflicted mitochondrial damage (84). Protecting mitochondrial function by curbing ROS generation can consequently mitigate target-organ injury. The mitochondrial iron–sulfur protein mitoNEET is an important modulator of mitochondrial oxidative capacity; its inhibition with NL-1 or mitoNEET shRNA abrogates LPS-induced ROS production and mitochondrial dysfunction (85). Uncoupling protein 2 (UCP2) lowers ROS via proton leak (86). In septic cardiomyopathy, UCP2 prevents endotoxin-induced oxidative stress and apoptosis in cardiomyocytes (87) and, by preserving mitochondrial morphology and function, also protects against LPS-induced acute kidney injury (88). Up-regulating the mitochondrial deacetylase sirtuin-3 (SIRT3) suppresses oxidative stress, safeguards mitochondrial integrity, and promotes autophagy in intestinal epithelial cells, thereby attenuating sepsis-related intestinal injury (89). Maintaining mitochondrial homeostasis—balancing fusion/fission and biogenesis—shields lung and kidney tissue from sustained oxidative stress (90).

Mitochondria are central to regulated cell death, and damage occurs even in non-severe sepsis. Altered mitochondrial membrane potential and assembly of the mitochondrial permeability-transition pore (MPT) drive necrosis (91) and apoptosis (92). During sepsis-related apoptosis, stressed or injured mitochondria release cytochrome c, triggering lymphocyte and epithelial-cell death (93). Clinicopathological study in patients with severe sepsis indicate that extensive lymphocyte apoptosis promotes shock and multiple-organ-dysfunction syndrome (MODS) and exacerbates the immune dysfunction typical of the immunoparalytic phase (94). In septic mice, diaphragmatic mitochondria show increased proton leak and superoxide-dismutase-2 (SOD2) down-regulation, changes linked to ROS-mediated apoptosis and ferroptosis (95). Among several injury pathways, iron-dependent damage is crucial because it underpins cascades that intensify tissue injury. Sepsis enhances lipid peroxidation, especially ferroptosis. Transferrin receptor 1 (TFR1) up-regulation and increased divalent metal transporter 1 (DMT1) activity raise intracellular iron (96); macrophages likewise release iron after phagocytosing damaged erythrocytes (97). ROS accumulation and expansion of the labile iron pool foster ferroptosis, a necrotic death that liberates DAMPs (98) and amplifies inflammation. Inflammasome formation and caspase activation cleave gasdermin; the N-terminal fragment forms membrane pores and induces pyroptosis (99, 100). Yes-associated protein 1 (YAP1) restrains ferroptosis by modulating the labile iron pool. YAP1-knock-out septic mice develop more severe acute-lung injury and display weaker ferroptosis defenses—glutathione peroxidase 4 (GPx4), TH1 and SLC7A11 are down-regulated, whereas pro-peroxidation factors SFXN1 and NCOA4 rise (101). In LPS-stimulated pulmonary epithelial cells, YAP1 loss enlarges the labile iron pool and disrupts mitochondrial function, initiating ferroptosis, whereas YAP1 over-expression blocks Fe³^+^-to-Fe²^+^ conversion and preserves mitochondrial integrity (101).

Mitochondria also play key roles in innate immunity. One mechanism is inflammasome activation (102). During infection, mitochondrial ROS rise in immune and non-immune cells responding to PAMPs (103) and DAMPs (104). Although ROS help eradicate pathogens—especially bacteria—excess levels damage mitochondria and tissues (105). PAMPs and DAMPs stimulate the NLRP3 inflammasome (106, 107). mtDNA is a critical DAMP driving immune responses and injury (108). Once released from damaged or stressed cells, mtDNA enters neighboring cells and activates the inflammasome by binding NLRP3 (109), initiating production of IL-1β and IL-18 (110). These cytokines propagate systemic inflammation, leading to tissue damage and organ dysfunction in sepsis (111). Translational study in human sepsis suggest that early recovery of mitochondrial biogenesis in leukocytes can reverse initial mitochondrial injury and is associated with improved clinical outcomes (112). The mitochondrial antiviral signaling protein serves as a hub for RIG-I-like-receptor pathways against RNA viruses (113). Mitochondrial DAMPs—mtDNA among them—activate innate immunity via TLR-9, the NLRP3 inflammasome and cGAS–STING signaling (114).

Metabolic intermediates further shape mitochondrial regulation of innate immunity. Itaconate (ITA), an anti-inflammatory Krebs-cycle metabolite, is produced in high amounts in LPS-stimulated macrophages via rapid induction of the mitochondrial enzyme IRG1. Cytosolic ITA alkylates transcription factor EB (TFEB), driving its nuclear translocation; nuclear TFEB enhances lysosome biogenesis and augments bacterial clearance (115). ITA also competitively binds α-ketoglutarate and inhibits ten eleven translocation (TET) DNA dioxygenases, modulating inflammatory-gene expression and identifying TET2 as a key anti-inflammatory ITA target (116). As a central metabolic organelle, mitochondria thus critically shape immunometabolism; manipulating mitochondrial function to recalibrate immune cells metabolism may offer new avenues for restoring immune homeostasis in sepsis.

This section underscores that mitochondria lie at the core of sepsis pathophysiology by coupling bioenergetics, redox balance and innate immunity. Redox-driven dysfunction of the mitochondrial network—shaped by regulators such as mitoNEET, UCP2, SIRT3 and the balance of fusion, fission and biogenesis—determines the extent of organ protection versus injury by influencing ROS production, apoptosis, ferroptosis and pyroptosis. Iron-dependent lipid peroxidation, expansion of the labile iron pool and loss of YAP1-mediated control promote ferroptosis and DAMP release, amplifying inflammatory damage. At the same time, mitochondrial ROS and DAMPs, especially mtDNA, drive NLRP3 inflammasome activation and systemic cytokine release, while metabolites such as itaconate feedback on lysosome biogenesis and inflammatory-gene regulation, highlighting mitochondria as central hubs of immunometabolism and attractive targets for restoring immune homeostasis in sepsis.

## 4 Cell-specific metabolic rewiring

### 4.1 Macrophage immunometabolism

Exposure of macrophages to pathogen-derived components such as LPS, lipoteichoic acid (LTA) and peptidoglycan (PGN), or to cytokines including tumor-necrosis factor-α (TNF-α), interleukin-1 (IL-1), IL-4 and IL-10, triggers marked metabolic reprogramming (117). Energy production shifts from OXPHOS to glycolysis, allowing rapid ATP synthesis to meet the abrupt demand generated by pathogen or inflammatory stimuli (**Table 1**) (118). During LPS stimulation, enhanced glycolysis is accompanied by up-regulation of glucose transporter-1 (GLUT1), thereby satisfying the increased glucose requirement of glycolytic metabolism (119). In prolonged inflammatory states such as sepsis, this metabolic switch is essential for macrophage survival (120), and it reshapes gene-expression programs that determine pro- and anti-inflammatory functions (121).

**Table 1:** Summary map of immunometabolic pathways, principal cell types, key mediators/signals, and major functional effects.

Metabolic pathway	Main cell type	Key mediator/signal	Main effect	Model	Reference
Metabolic switch from OXPHOS to glycolysis	Macrophages	LPS	Accelerates ATP generation to meet heightened bioenergetic demand during inflammation	–	(118)
Glycolytic upregulation with increased glucose uptake	Macrophages	GLUT1 upregulation	Enhances glucose import to sustain glycolytic flux	Murine peritoneal macrophages	(119)
Pro-inflammatory metabolic program	M1 macrophages	HIF-1α; mTORC1	Supports pro-inflammatory effector output	Mouse macrophages	(123, 124)
Upstream activation of glycolysis-associated signaling	M1 macrophages	BCAP; PI3K; Akt; mTORC1	Increases glucose uptake and utilization capacity	RAW264.7 cells and BCAP^−/−^ mouse macrophages	(125, 126)
Metabolite-driven amplification of inflammatory signaling	M1 macrophages	Succinate accumulation; HIF-1α stabilization	Promotes IL-1β release and exacerbates inflammatory responses	Mouse bone-marrow–derived macrophages and LPS-treated mice	(128–130)
Glycolytic enzyme involvement in pro-inflammatory transcriptional control	M1 macrophages	PKM2; HIF-1α	Facilitates IL-1β production and release	LPS-stimulated mouse macrophages	(131)
Inflammasome pathway restraint	M1 macrophages	PKM2 deficiency	Suppresses NLRP3 and AIM2 inflammasomes and alleviates sepsis severity	Pkm2^−/−^ mice and derived macrophages	(63)
Predominant fatty acid oxidation and mitochondrial oxidative metabolism	M2 macrophages	FAO; mitochondrial OXPHOS	Sustains immunosuppressive functions	IL-4–polarized mouse M2 macrophages	(134, 135)
Predominant fatty acid oxidation	Thymus-derived Tregs	Low GLUT1 expression; FAO	Relies primarily on FAO for energy supply	Mouse thymic Tregs *in vitro*/*in vivo*	(143)
Mitochondrial oxidative metabolism supporting suppressive function	Tregs	ROS; NFAT; Foxp3 CNS2	Reinforces Foxp3^+^ Treg stability and maintains suppressive capacity	Mouse Tregs in transplantation/autoimmunity models	(144, 146, 147)
Enhanced fatty acid uptake and FAO	Tregs	PPAR signaling; CD36	Augments suppressive activity and is associated with worse sepsis outcomes	Mouse Tregs in sepsis models	(149)
Promotion of OXPHOS	Tregs	Metformin; AMPK	Strengthens immunosuppressive activity and contributes to impaired immune responsiveness	Mouse T cells; metformin-treated disease models	(150, 151)
Reduced glycolysis with translational repression of TNF-α	Macrophages	GAPDH binding to TNF-α mRNA	Contributes to an immunoparalysis phenotype; increased glycolysis can reverse this state	Human monocyte/macrophage models under low glycolysis	(64)
Lactate-associated reinforcement of immunosuppression	Tregs	Increased lactate; increased PD-1	Further intensifies immunosuppression	Tumor-infiltrating Tregs from mice and patients	(141)

**Figure 4:**
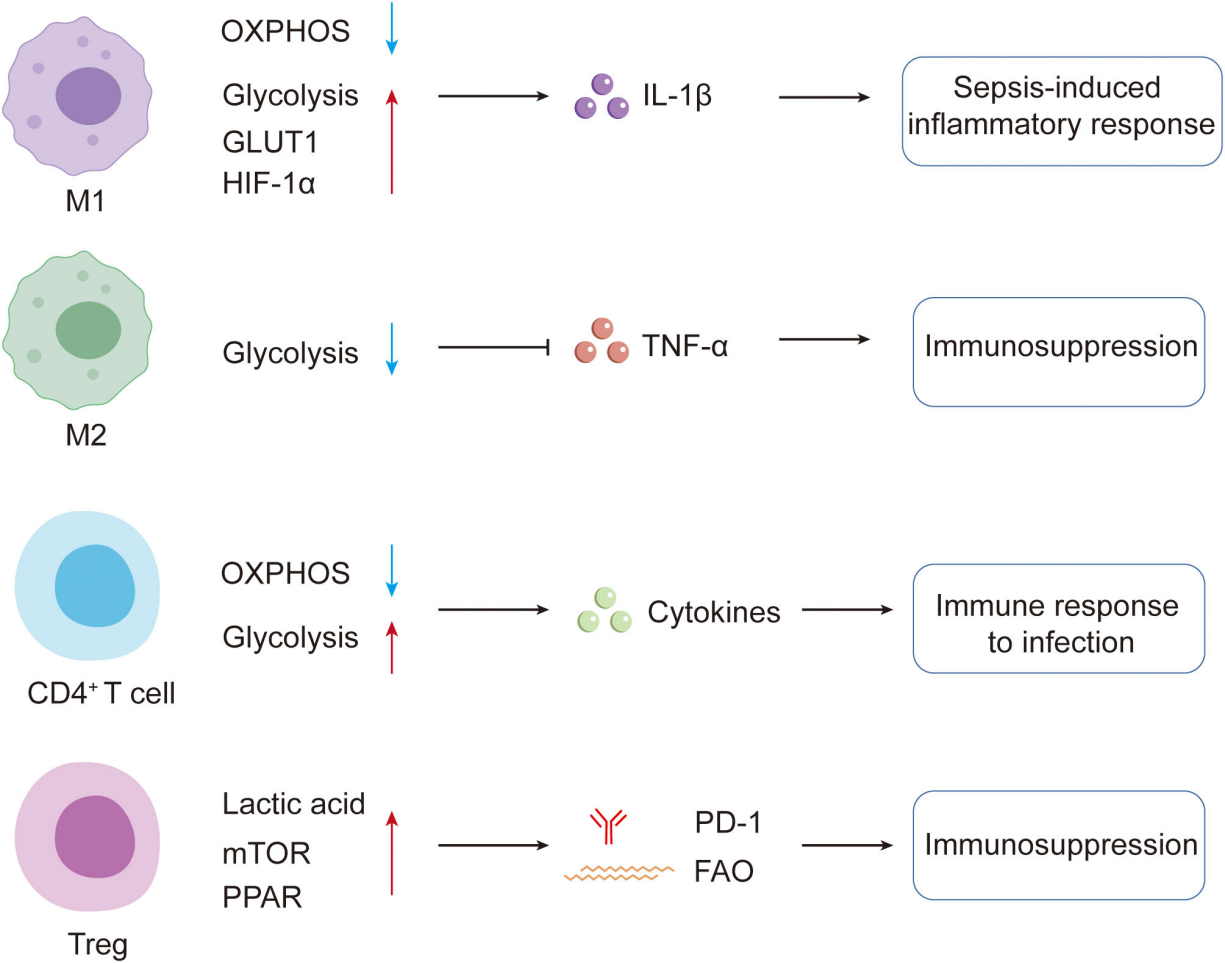
Infection-driven immunometabolic reprogramming of macrophages and T cells in sepsis. Infection-triggered acute inflammatory immune responses can drive the differentiation of undifferentiated monocytes/macrophages towards classically activated M1 macrophages, a process accompanied by up-regulation of GLUT1 and a metabolic shift from OXPHOS to glycolysis. In addition, HIF-1α induced during LPS stimulation of macrophages promotes increased release of IL-1β, thereby exacerbating the inflammatory response in sepsis. By contrast, M2 macrophages, characterized by lower glycolytic activity, can suppress expression of TNF-α and contribute to immunosuppressive programs. Upon activation, CD4^+^ T cells undergo a metabolic transition from OXPHOS to aerobic glycolysis and participate in antimicrobial immunity by promoting cytokine expression. In Tregs, elevated lactate enhances expression of PD-1, further aggravating immunosuppression. Moreover, during sepsis the mechanistic target of mTOR signaling pathway can drive activated Tregs towards a more suppressive state, whereas PPARs potentiate Treg suppressive function by promoting FAO; together, these mechanisms contribute to adverse outcomes in sepsis. GLUT1, glucose transporter 1; OXPHOS, oxidative phosphorylation; HIF-1α, hypoxia-inducible factor-1α; LPS, lipopolysaccharide; IL-1β, interleukin-1β; TNF-α, tumor necrosis factor-α; Tregs, regulatory T cells; PD-1, programmed cell death protein 1; mTOR, mechanistic target of rapamycin; PPARs, peroxisome proliferator-activated receptors; FAO, fatty acid oxidation.

Acute inflammatory responses convert undifferentiated monocytes/macrophages into classically activated (M1) macrophages, which are pivotal during sepsis (122). M1 cells secrete large amounts of pro-inflammatory mediators to eradicate pathogens, and this function depends on the metabolic transition from OXPHOS to glycolysis (**Figure 4**) (123, 124). The switch is mediated by HIF-1α and mammalian target of mTORC1 (123). Under LPS challenge, B-cell adaptor for PI3K (BCAP) activates phosphatidylinositol-3-kinase, which in turn stimulates Akt and mTORC1 (125, 126). Akt also enhances endosomal recycling of GLUT1, increasing its surface expression (127) and thereby augmenting glucose uptake and utilization in M1 macrophages. Intracellular succinate accumulation during LPS stimulation further stabilizes HIF-1α, driving IL-1β release (128, 129). Thus, succinate directly amplifies HIF-1α-mediated inflammatory pathways in M1 cells and exacerbates sepsis-associated inflammation (130). The glycolytic enzyme PKM2 reinforces IL-1β production in LPS-stimulated M1 macrophages (131). Myeloid-specific PKM2 deletion alleviates sepsis by suppressing NLRP3 and AIM2 inflammasomes (63). HIF-1α also induces pyruvate-dehydrogenase-kinase 1 (PDK1), further promoting glycolysis and enhancing macrophage migration to sites of inflammation or injury (132). Conversely, macrophages lacking HIF-1α display defective M1 activation: glycolytic genes are poorly induced, glucose uptake and metabolism are limited, and production of pro-inflammatory cytokines such as IL-1β is impaired (133).

Alternatively activated (M2) macrophages, which possess immunosuppressive functions, play major roles in parasitic infection, tissue remodeling and tumor progression (134). M2 cells rely primarily on FAO and mitochondrial OXPHOS for energy (135). *De-novo* fatty-acid synthesis supplies substrates for FAO and supports their immunosuppressive phenotype (136). M2 macrophages can redirect glucose to drive OXPHOS and reprogrammed toward the M1 phenotype, whereas M1 cells cannot revert to M2 because mitochondrial OXPHOS is suppressed (137). Low glycolytic flux permits glyceraldehyde-3-phosphate dehydrogenase to bind TNF-α mRNA and inhibit its translation (64), a key feature of late-stage immunoparalysis; boosting glycolysis can reverse this state (64).

### 4.2 Regulatory T-cell energetic remodeling

**Figure 5:**
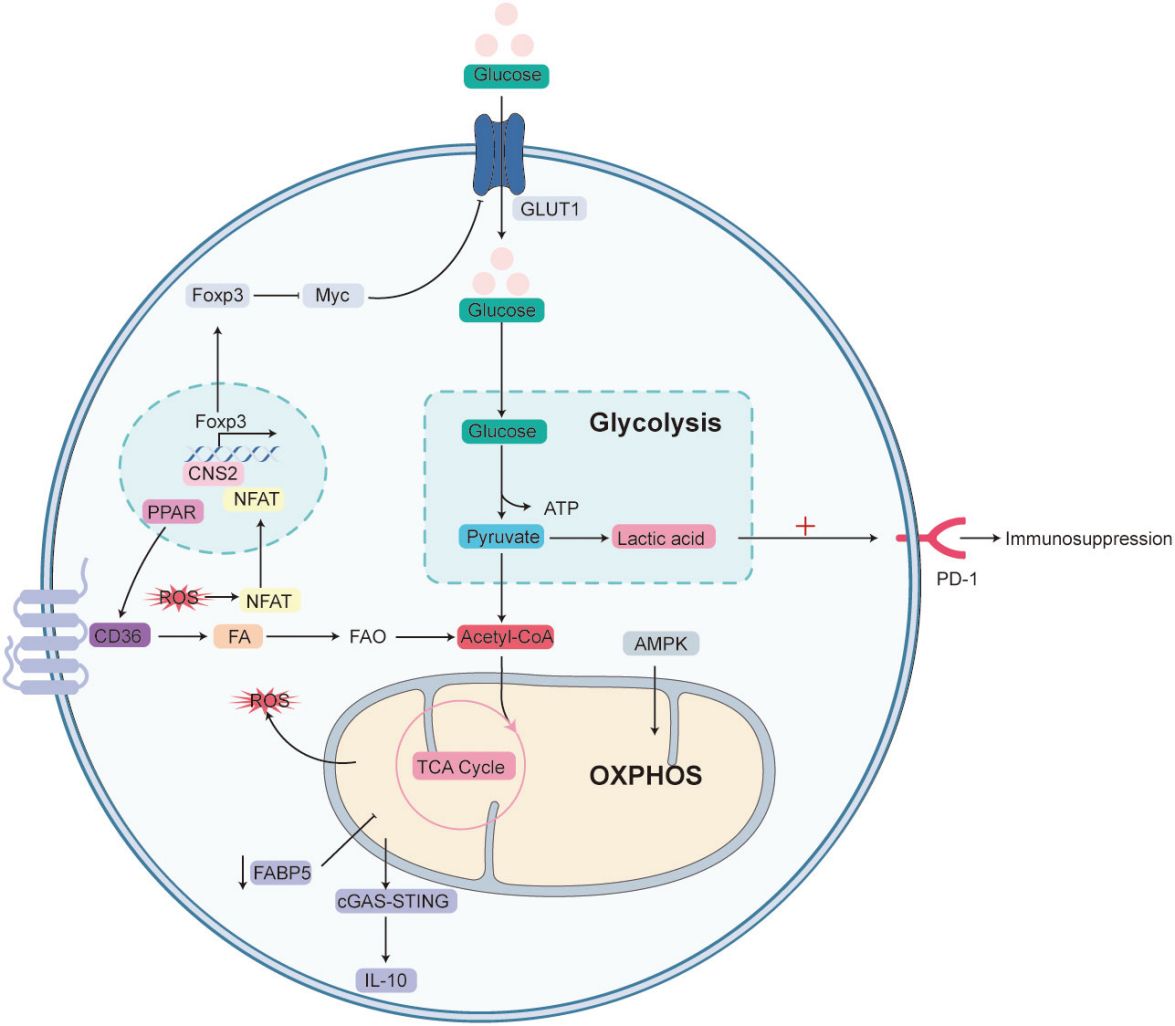
Immunometabolic dynamics shape the immunosuppressive capacity of Tregs. Under homeostatic conditions, Foxp3 suppresses glycolysis by down-regulating Myc and shifts metabolism toward mitochondrial oxidative phosphorylation (OXPHOS), thereby increasing Tregs’ resistance to lactate-induced proliferative arrest. Persistent lactate accumulation further up-regulates PD-1 on the Treg surface, reinforcing their suppressive activity. Thymus-derived Tregs (tTregs), which express low levels of the glucose transporter GLUT1, rely primarily on CD36-mediated fatty-acid uptake followed by fatty-acid oxidation (FAO) for energy production. Within mitochondria, OXPHOS-derived reactive oxygen species (ROS) facilitate binding of NFAT to the CNS2 enhancer of the Foxp3 gene, stabilizing Foxp3 expression and consolidating the Treg phenotype. Concurrently, activation of PPAR further increases CD36 expression, amplifying fatty-acid uptake and FAO and thus enhancing immunosuppressive function; activation of AMPK likewise promotes OXPHOS, helping maintain suppressive properties. In contrast, inhibition of fatty-acid-binding protein 5 (FABP5) impairs mitochondrial function, lowers OXPHOS and disrupts lipid homeostasis, which triggers cGAS–STING-dependent IFN-I signaling, elevates IL-10 production and further strengthens Treg-mediated suppression.

Tregs constitute ~5–10% of mouse CD4^+^ T cells and ~5% of their human counterparts (138). Accumulating evidence indicates that Tregs are not a single entity but comprise multiple subsets with distinct functions and surface markers (139). In activated CD4^+^ T cells, metabolism is rewired toward aerobic glycolysis, which is regarded as an initial step in host anti-infection immunity (30). In Tregs, however, the pattern is reversed: under physiological conditions, Foxp3 suppresses Myc and glycolysis while promoting OXPHOS, thereby rendering Tregs resistant to lactate-induced proliferation arrest (**Figure 5**) (140). Pathophysiologically, lactate up-regulation enhances programmed cell death protein-1 (PD-1) expression on Tregs and deepens immunosuppression (141). An increasing number of metabolites promote Treg proliferation and migration (142). Unlike conventional CD4^+^ T cells, thymus derived Tregs (tTregs) express low levels of GLUT1 and therefore rely mainly on FAO, rather than glycolysis, for energy (143); ex-vivo analyses show increased mitochondrial mass and enhanced function in tTregs (144).

Although glycolysis supports Treg proliferation and differentiation, oxidative metabolism is critical for their suppressive activity (145). Mitochondrial oxidation is closely linked to ROS production, which stabilizes Foxp3^+^ Tregs by promoting binding of nuclear factor of activated T cells (NFAT) to the CNS2 enhancer of the Foxp3 locus (144, 146, 147). During sepsis, mammalian target of mTOR signaling drives activated Tregs toward a more suppressive phenotype (148). Peroxisome proliferator-activated receptor (PPAR) signaling enhances fatty-acid uptake via CD36 and boosts FAO, augmenting Treg suppression and contributing to poor sepsis outcomes (149). Conversely, metformin indirectly activates AMP-activated protein kinase, promotes OXPHOS and, by reinforcing Treg suppression, worsens immune-response defects (150, 151). Dynamic lipid metabolism is considered central to Treg activation and sustains their survival and function. Fatty acid binding proteins (FABPs) facilitate lipid uptake and intracellular trafficking; inhibition of FABP5 impairs mitochondrial function, lowers OXPHOS and disrupts lipid homeostasis, thereby activating cGAS–STING-dependent type I interferon signaling, increasing IL-10, and intensifying Treg suppression (152). In sum, glycolysis favors Treg migration, FAO supports proliferation, and OXPHOS regulators are pivotal for maintaining suppressive function (152). Targeting oxidative metabolism may destabilize Foxp3^+^ Tregs and enhance host antimicrobial immunity during sepsis-induced immunosuppression.

### 4.3 Temporal and pathogen-dependent immunometabolic heterogeneity in sepsis

#### 4.3.1 Temporal transition from early hyperinflammation to late immunosuppression

**Figure 6:**
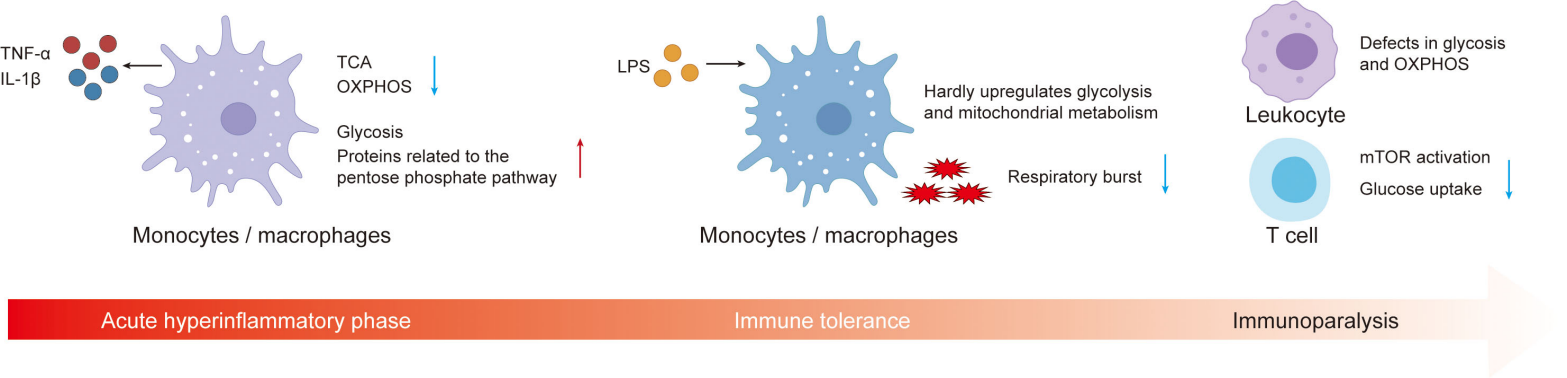
Temporal immunometabolic remodeling of monocytes, macrophages, and T cells in sepsis. During the acute hyperinflammatory phase, peripheral monocytes and tissue macrophages undergo metabolic reprogramming from mitochondrial oxidative phosphorylation to aerobic glycolysis, accompanied by downregulation of the tricarboxylic acid cycle and fatty acid oxidation, in order to meet the energetic demands for the synthesis of proinflammatory mediators such as TNF-α and IL-1β. Proteins involved in glycolysis and the pentose phosphate pathway are markedly upregulated. Repeated LPS exposure induces an immune-tolerant monocyte phenotype in which cells are almost unable to further upregulate glycolysis and mitochondrial metabolism upon restimulation, and their oxidative burst is markedly attenuated, indicating that loss of metabolic plasticity directly compromises effector function. Transcriptomic and metabolomic analyses of patients with sepsis in the immunoparalysis phase reveal widespread defects in both glycolysis and oxidative phosphorylation in peripheral leukocytes. At the T-cell level, patients with septic shock exhibit immunometabolic failure characterized by impaired mTOR activation and reduced glucose uptake.

Sepsis is not a single disease entity but a syndrome characterized by marked heterogeneity in immune phenotypes and clinical outcomes; even within an individual patient, the disease course may encompass distinct stages, evolving from early hyperinflammation to subsequent immunosuppression (153, 154). During the acute hyperinflammatory phase, peripheral monocytes and tissue macrophages undergo metabolic reprogramming from mitochondrial oxidative phosphorylation to aerobic glycolysis, accompanied by downregulation of the tricarboxylic acid (TCA) cycle and fatty acid oxidation, thereby meeting the energy requirements for the synthesis of proinflammatory cytokines such as TNF-α and IL-1β (**Figure 6**) (30, 58, 155). Proteomic analyses of peripheral blood mononuclear cells from patients with sepsis further demonstrate that, compared with healthy controls, proteins involved in glycolysis and the pentose phosphate pathway are markedly upregulated, whereas multiple mitochondrial energy metabolism proteins are downregulated or exhibit functional alterations. These findings indicate that, already at early stages, an abnormal network characterized by enhanced glucose metabolism and mitochondrial dysfunction is established (156). In human endotoxemia models, repeated LPS exposure induces an immune-tolerant monocyte phenotype in which cells are almost unable to further upregulate glycolysis and mitochondrial metabolism upon restimulation. This loss of metabolic plasticity is associated with impaired oxidative burst and reduced Candida albicans killing, directly linking defective metabolic adaptability to compromised effector function (157). In patients with sepsis who have entered the immunoparalysis phase, integrated transcriptomic and metabolomic analyses reveal broad defects in both glycolysis and oxidative phosphorylation in peripheral leukocytes; these abnormalities are only partially reversed after clinical recovery, suggesting that disturbed energy metabolism is a key basis of late immunosuppression (30). At the T-cell level, patients with septic shock exhibit immunometabolic failure characterized by impaired mTOR activation and reduced glucose uptake. *In vitro*, recombinant IL-7 restores mTOR activation, upregulates GLUT1 expression, and enhances T-cell glucose uptake and proliferation, thereby partially reversing these functional defects (158). Collectively, these observations support a model in which early sepsis is dominated by a hyperinflammatory response driven by enhanced glycolysis and mitochondrial injury, whereas with disease progression, loss of metabolic plasticity, insufficient mitochondrial energy supply, and lymphocyte exhaustion become prominent immunosuppressive phenotypes. These temporal and endotype-dependent differences have been systematically summarized in recent reviews on sepsis immunometabolism and mechanisms of immunosuppression, providing a conceptual basis for stage-adapted, individualized immunometabolic interventions.

#### 4.3.2 Pathogen category–dependent immunometabolic reprogramming in bacterial, viral, and fungal sepsis

The category of the causative pathogen is a major determinant of immune phenotype and metabolic state in sepsis; bacterial, viral, and fungal sepsis differ substantially in incidence, prognosis, and inflammatory mediator profiles (159). Integrating clinical and experimental data on microbial sepsis, in predominantly bacterial sepsis monocytes and macrophages rapidly shift from mitochondrial oxidative phosphorylation to high-rate glycolysis, accompanied by accumulation of lactate and succinate and increased production of itaconate. These metabolites amplify early inflammatory responses by stabilizing HIF-1α and promoting IL-1β transcription (160). As the disease progresses, depletion of energetic substrates and mitochondrial damage further reduce ATP generation and constrain oxidative metabolism, which coincides with peripheral T-cell functional failure and the emergence of an immunosuppressive phase (11). Severe COVID-19–associated disease represents a prototypical form of viral sepsis. In monocytes, SARS-CoV-2 infection induces HIF-1α–dependent enhancement of aerobic glycolysis and lactate accumulation, which not only drives the release of proinflammatory cytokines such as IL-1β but also provides carbon sources and energy to support viral replication (161). Patients with pre-existing metabolic syndrome, including obesity and type 2 diabetes, already exhibit chronic low-grade inflammation and energy metabolic imbalance at baseline. This metabolic background is closely associated with an increased risk of cytokine storm and multiple organ failure, indicating that immunometabolic reprogramming in viral sepsis is more readily amplified by prior host metabolic derangements (162). In contrast, in fungal sepsis, host metabolic stress more prominently manifests as disruption of systemic glucose homeostasis and competition for carbon sources between host and pathogen. In murine intravenous Candida infection models, Candida consumption of glucose in the bloodstream and organs leads to host hypoglycemia and impairs the survival and fungicidal capacity of glycolysis-dependent macrophages (163). Further studies confirm that competition for glucose between host macrophages and Candida finely tunes NLRP3 inflammasome activation and IL-1β production, and that modulating glucose metabolic pathways can significantly alter the magnitude of inflammation associated with fungal infection (164). Murine fungal sepsis models demonstrate that systemic disturbances in glucose metabolism and hypoglycemia are very common, and that pharmacologic interventions targeting host glucose metabolism with 2-deoxyglucose or metformin worsen the outcome of fungal infection and exacerbate hypoglycemia (165). Taken together, these studies suggest that bacterial sepsis is characterized by a short-lived burst of high glycolytic activity followed by secondary energetic failure; in viral sepsis, immune cells glycolysis both participates in antiviral responses and may be exploited by the virus; and in fungal sepsis, outcomes depend more heavily on prolonged metabolic competition between host and pathogen for glucose and other energetic substrates. These distinct patterns collectively shape a differential landscape of immunometabolic reprogramming across pathogen categories.

## 5 Targeting metabolic nodes: emerging therapeutic strategies

Targeting the immunometabolism of immune cells at specific sepsis stages is increasingly recognized as crucial, and interventions aimed at the metabolic programs of macrophages and T cells have emerged as novel strategies (166). Recent agents that modulate immune cells metabolism in sepsis are summarized in **Table 2**.

**Table 2:** Immunometabolic modulators and their therapeutic roles in sepsis.

Therapeutic agent	Target	Experimental model	Key effects	Reference
Plumbagin	PKM2	Balb/C mice	Inhibit LPS-induced aerobic glycolysis by downregulating the expression of pyruvate kinase M2, limit proinflammatory cytokine (IL-1β and HMGB1) release in macrophages, protect mice from lethal endotoxemia and polymicrobial sepsis.	(178)
Shikonin	PKM2	C57BL/6J mice	Target PKM2 to inhibit lactate production and HMGB1 release, enhance the survival rate of AMPK-α1-deficient septic mice.	(179)
AICAR	AMPK	C57BL/6 mice	Limit CLP and LPS-induced upregulation of intracellular adhesion molecule, decrease inflammatory cytokines.	(181)
A-769662	AMPK	C57BL/6J mice	Activate AMPK, reduce HMGB1 release and septic death in AMPKα-deficient mice.	(179)
Metformin	AMPK, PGC-1α	C57BL/6J mice	Enhance the expression of p-AMPK and PGC-1α, inhibit HIF-1α expression and downstream inflammatory mediators, HMGB1 and TNF-α, attenuate inflammation and liver injury in septic aged mice.	(184)
	AMPK	C57BL/6 mice	Increase mitochondrial complex V function and amounts of ETC complex III and IV, reduces the severity of polymicrobial sepsis-induced lung injury and prevents the development of sepsis-associated immunosuppression.	(183)
Dimethyl malonate	Succinate oxidation	C57BL/6 mice	Inhibit macrophage succinate oxidation exerts anti-inflammatory effects in sepsis	(187)
rhIL-7	mTOR	T lymphocyte	Improve mTOR activation, GLUT1 expression, and glucose entry in septic patients’ T lymphocytes, leading to their enhanced proliferation.	(158)
CYT107	–	Patients with septic shock and severe lymphopenia	Reverse the loss of CD4+ and CD8+ immune effector cells, and restore adaptive immunity.	(189)

### 5.1 Immunometabolic endotypes of sepsis and metabolic marker–based stratification

According to the Sepsis-3 consensus and subsequent large cohort and multi-omics studies, sepsis is now recognized as a highly heterogeneous syndrome. Stratification based solely on infection source or conventional organ dysfunction scores is insufficient to fully account for prognostic heterogeneity, whereas metabolomic and lipidomic analyses have identified a series of metabolic endotypes centered on disordered energy metabolism, thereby establishing a more direct link between immunometabolic mechanisms and clinical phenotypes (167, 168). Untargeted metabolomics in multicenter cohorts of critically ill patients has shown that a subset of individuals with acute respiratory failure and infection-related critical illness exhibit a bioenergetic-dysfunction metabolic endotype, characterized by markedly elevated plasma short-chain and medium-chain acylcarnitines and reduced acyl-glycerophosphocholine levels; this pattern is strongly associated with increased risk of death and has been interpreted as a signature of mitochondrial and bioenergetic dysfunction (169). In multicenter adult sepsis cohorts, higher plasma acetylcarnitine levels are independently associated with higher multiple organ dysfunction scores and increased 28-day mortality, indicating that patients with elevated acetylcarnitine and related acylcarnitine abnormalities experience more severe organ dysfunction and inflammatory burden (168, 170). Among adults with severe sepsis and septic shock, elevated plasma cell-free mitochondrial DNA is associated with higher APACHE II scores and increased mortality risk; patients with high cf-mtDNA also display concomitant increases in short-chain acylcarnitines and decreases in glycerophosphocholine-related metabolites, supporting the concept of a metabolic endotype characterized by mitochondrial damage and abnormal lipid remodeling (171). In addition, studies in emergency department sepsis cohorts have shown that both capillary and serum lactate rise in parallel with short-term mortality, underscoring the clinical utility of lactate for identifying patients with higher metabolic load and worse prognosis (172).

At the immune cells level, the transition of the systemic immune response in septic shock from early hyperinflammation to later immunoparalysis is driven in part by the remodeling of immune cell metabolic programs, as systematically summarized in recent work on leukocyte function and immunometabolism (173). Single-cell transcriptomic analyses have shown that, in a subset of patients with severe sepsis and septic shock, peripheral blood monocytes form clusters characterized by enhanced glycolysis and relatively attenuated mitochondrial oxidative phosphorylation; in these clusters, glycolysis-related gene modules correlate closely with HIF1A expression, alongside up-regulation of inflammation-related pathways and down-regulation of HLA class II antigen-presentation modules, consistent with immune cells metabolic endotypes defined by glycolytic reprogramming and mitochondrial dysfunction (174). At the level of circulating metabolites, persistent hyperlactatemia not only reflects tissue hypoperfusion but is also closely linked to immunosuppression. Lactate, via monocarboxylate transporters and GPR81-mediated signaling, reprograms the metabolism and function of multiple immune cell types and can induce lactylation and acetylation of histones and proteins such as HMGB1, thereby reshaping macrophage inflammatory phenotypes and influencing T-cell effector function; these mechanisms are supported by data from various sepsis animal models and human immune cell studies (175, 176). A single-center prospective adult intensive care study further showed that baseline serum levels of the ferroptosis-related proteins ACSL4, GPX4 and PTGS2 are significantly higher in sepsis patients than in non-septic critically ill patients and healthy controls, and that elevated ACSL4 is closely associated with higher SOFA scores, a greater incidence of septic shock and increased 28-day mortality, suggesting that ferroptosis-related markers may help identify patient subgroups with higher ferroptotic activity (177). In a mouse model of sepsis-induced acute lung injury, YAP1 suppresses ferroptosis by regulating the labile iron pool and modulating the expression of antioxidant factors such as GPX4 and SLC7A11, thereby attenuating lung tissue damage and providing mechanistic support for the clinical ferroptosis markers described above (101). Taken together, evidence from systems-level metabolomics, immune cells transcriptomics and functional studies suggests that, across different sepsis endotypes, immunometabolic abnormalities such as enhanced glycolysis, mitochondrial dysfunction, amplified lactate signaling, acylcarnitine accumulation, dysregulated lipid remodeling and ferroptosis-related lipid peroxidation may coexist in various combinations. These abnormalities can be leveraged for exploratory stratification in clinical and translational studies through measurable indicators including lactate, acylcarnitine profiles, lipidomic signatures, cf-mtDNA and ferroptosis-related proteins, thereby providing a candidate biomarker framework for future immunometabolism-based precision subtyping and targeted interventions.

### 5.2 Immunometabolic modulation strategies in sepsis and supporting clinical evidence

#### 5.2.1 Preclinical targeting of macrophage metabolic checkpoints

Zhang et al. reported that plumbagin—a naphthoquinone derivative from the root of *Plumbago zeylanica*—reduces PKM2 activity in macrophages, suppresses glycolysis and the release of IL-1β and HMGB1, and increases survival in septic mice (178). Likewise, the PKM2-targeting shRNA compound shikonin limits lactate production and HMGB1 release, improving survival in AMP-activated protein kinase α1 (AMPK-α1) deficient septic mice (179). In inflammatory settings, pro-inflammatory macrophages show diminished AMPK activity (180). The AMP analogue 5-aminoimidazole-4-carboxamide ribonucleoside (AICAR) activates AMPK and confers protection in murine sepsis (181); its benefit may stem from prompt AMPK-mediated control of macrophage immunometabolic reprogramming, which otherwise favors aerobic glycolysis via HMGB1-driven PKM2 activation (179). The direct AMPK activator A-769662 similarly suppresses HMGB1 release and mitigates inflammatory injury in septic mice (179). Metformin enhances AMPK and PGC-1α expression while down-regulating HIF-1α, HMGB1 and TNF-α, thereby protecting against sepsis-induced hepatic injury (182). AMPK activation by metformin preserves mitochondrial complex V (ATP synthase) activity, maintains electron-transport-chain complexes III and IV, and limits HIF-1α accumulation, thus strengthening innate immunity (183, 184). During sepsis, expression of isocitrate dehydrogenase 1 (IDH1) declines (185), leading to succinate and citrate build-up in macrophages (186). Mitochondrial succinate is oxidized by succinate dehydrogenase (SDH), generating ROS and stabilizing HIF-1α (187). Dimethyl malonate inhibits succinate oxidation and exhibits anti-inflammatory effects in sepsis (187). However, these metabolically targeted interventions largely remain confined to small animal experiments or early-phase clinical trials, with limited levels of evidence; in addition, substantial heterogeneity in the timing of administration, dosing regimens, and enrolled populations across studies makes it difficult to generalize the results to the broader sepsis population. Although agents such as AICAR, A-769662, metformin, and dimethyl malonate have shown certain immunometabolic modulatory and organ-protective effects at the mechanistic level and in small-scale studies, robust evidence is still lacking as to whether these effects can be translated into consistent benefits on hard endpoints such as survival. Overall, if patients are not stratified according to biomarkers or immunometabolic endotypes and uniform interventions are applied based solely on a single metabolic pathway, this is likely to be one of the key reasons why metabolically targeted therapies have repeatedly failed in previous clinical trials.

#### 5.2.2 Clinical immunometabolic interventions and trial outcomes

Septic patients often display profoundly reduced interleukin-7 (IL-7) concentrations, yet IL-7 signaling is essential for naïve T-cell survival and proliferation (188). Recombinant human IL-7 (rhIL-7) enhances mechanistic-target-of-mTOR signaling, GLUT1 expression and glucose uptake, thereby boosting T-cell proliferation (158). Francois and colleagues reported the IRIS-7 trial, a randomized, double-blind study in which patients with septic shock and marked lymphopenia were assigned to receive rhIL-7 or placebo. rhIL-7 significantly increased total peripheral lymphocyte counts and restored CD4^+^ and CD8^+^ T-cell numbers and function; however, in this small cohort no clear 28-day mortality benefit was demonstrated (189). Several small trials suggest that, in patients with profoundly reduced baseline mHLA-DR, GM-CSF can upregulate HLA-DR expression and restore peripheral monocyte TNF-α responses to LPS. Some studies also reported trends towards fewer secondary infections and improved organ dysfunction scores, yet effects on 28- or 90-day mortality have been inconsistent, largely owing to limited sample sizes and enrolment heterogeneity (190). Similarly, early phase I/II studies of PD-1 or PD-L1 blockade in sepsis indicate acceptable safety under intensive monitoring and show immunological signals, including reduced exhaustion-marker expression and enhanced T-cell proliferation and cytokine production. Nonetheless, these studies remain underpowered and largely focused on immunological endpoints, with no definitive evidence to date of improved long-term survival or recovery from multiple organ dysfunction (191).

As a representative metabolic modulator, vitamin C, either alone or combined with thiamine and glucocorticoids, has attracted substantial interest, with the aim of ameliorating macrophage and endothelial metabolic dysregulation through antioxidant and mitochondria-protective effects, thereby mitigating organ dysfunction. Early, small phase I studies suggested that high-dose intravenous vitamin C was generally well tolerated in critically ill patients with sepsis (192). However, randomized trials in ICU patients with sepsis did not improve clinical outcomes; notably, the primary composite endpoint was higher, with an increased proportion of 28-day death or persistent organ dysfunction, underscoring the potential risks of strategies predicated solely on antioxidant or redox-based intervention (193). Building on these data, a network meta-analysis integrating multiple randomized controlled trials of vitamin C, thiamine, and glucocorticoids found no robust benefit for long-term mortality, whether vitamin C was administered alone or in combination. Only modest improvements in SOFA score or duration of vasopressor use were observed in some studies, and the overall findings were highly heterogeneous (194). Likewise, anti-cytokine therapies targeting TNF and IL-1 have repeatedly failed to reduce overall mortality in large phase III trials, despite signals of attenuated inflammation and potential organ protection in preclinical models and in certain hyperinflammatory subgroups (153, 195).

Taken together, the repeated lack of clinical success with these therapies is closely related to the fact that most trials have been small, early-phase studies that rely predominantly on immunological or biomarker endpoints rather than hard outcomes such as survival (189, 191, 192). At the same time, sepsis is highly heterogeneous, and most trials have not implemented standardized stratification based on markers of immune exhaustion or immunometabolic endotypes, leading to substantial dilution of potential treatment effects in responsive subgroups (153, 190, 195). In addition, key biomarkers such as mHLA-DR lack assay standardization, and inclusion criteria and outcome assessments differ between centers, further exacerbating inconsistency in trial results (190). Network meta-analyses further indicate marked variation in dosing, treatment duration, and co-administration regimens with thiamine and corticosteroids, rendering conclusions regarding long-term mortality both non-robust and highly heterogeneous (193, 194). Overall, unless patient heterogeneity, endotype-based stratification, and trial design are more rigorously addressed, immunologic or metabolic effects observed in mechanistic and small-scale studies are unlikely to be translated into consistent survival benefits in large patient populations.

## 6 Conclusions and future perspectives

Sepsis is a systemic inflammatory syndrome initiated by dysregulated immunometabolism, and its high lethality is closely linked to metabolic reprogramming in immune cells. This review delineates three principal metabolic derangements that shape the disease course. (i) Lipid‐metabolic disruption, characterized by impaired cholesterol homeostasis and FAO impairment, amplifies organ injury. (ii) Early glycolytic hyper-activation represents the dominant carbohydrate abnormality. (iii) Mitochondrial collapse triggers uncontrolled ROS surges and cell-death signaling. Together, these disorders drive immune cells along a trajectory from hyper-inflammation to profound immunoparalysis and remodel host defenses through epigenetic mechanisms such as short-chain-fatty-acid–mediated histone acetylation. Targeting key nodes in these pathways shows therapeutic promise: inhibiting glycolytic enzymes curbs pro-inflammatory mediator release, modulating tricarboxylic-acid-cycle enzymes limits ROS and cytokine production, and enhancing T-cell glucose metabolism boosts lymphocyte proliferation and improves survival.

Current metabolic interventions can exert off-target effects on non-immune tissues, underscoring the need for delivery systems that home to specific organs or cell populations. Therefore, a key research priority going forward is to develop metabolic modulation strategies and drug delivery systems that can be targeted to specific organs and cell populations, thereby minimizing toxicity while maximizing therapeutic benefit. At present, evidence for immunometabolic reprogramming in immune cells comes mainly from murine and *in vitro* models, and human immune cells may differ substantially from their murine counterparts in both metabolic programs and responses to interventions. There is an urgent need to conduct metabolomics-centered, systematic studies in cohorts of patients with sepsis, using longitudinal sampling and multi-omics integration to precisely identify distinct immunometabolic endotypes and correlate these metabolic features with organ dysfunction and clinical outcomes, thereby enabling metabolomics-guided patient stratification and optimization of intervention timing. The marked heterogeneity of sepsis further mandates personalized strategies. Integrating metabolomics to define discrete metabolic endotypes and exploring synergy between metabolic modulation and immunotherapies are priority areas. Finally, multi-dimensional mapping of the metabolism–immunity interface may yield new, precisely targeted avenues for sepsis management. In addition, it will be important to integrate immunometabolic phenotypes with ICU nutritional support and immunotherapeutic strategies, thereby constructing a comprehensive intervention framework centered on metabolism–immunity–nutrition. By dissecting the metabolism–immunity interaction network in a multidimensional manner, it may be possible to develop more precisely targeted and individualized therapeutic approaches for sepsis.
